# EHMTI-0265. Premonitory symptoms in episodic migraine: a multicenter questionnaire study of serbian headache society

**DOI:** 10.1186/1129-2377-15-S1-D47

**Published:** 2014-09-18

**Authors:** A Radojicic, S Sretenovic, D Rakic, A Mitrovic, A Stanic, S Sakac, S Simic, N Sternic, J Zidverc-Trajkovic

**Affiliations:** 1 Headache Center, Neurology Clinic, Belgrade, Serbia; 2 Migraine Center, Clinical Center “Zvezdara”, Belgrade, Serbia; 3 Neurology department, General Hospital Uzice, Uzice, Serbia; 4 Headache Center, Neurology Clinic, Novi Sad, Serbia

## Introduction

The International Classification of Headache Disorders defines premonitory symptoms as symptoms preceding and forewarning of a migraine attack by 2-48 h, occurring before the aura in migraine with aura and before the onset of pain in migraine without aura. Prevalence rates of patients reporting one or more premonitory symptoms range between 33% and 79% in clinic-based studies.

## Aims

The aim of our study was to evaluate the occurrence and characteristics of premonitory symptoms, and compare them between two migraine subtypes-with and without aura.

## Methods

A multicenter study under the auspices of Serbian Headache Society was conducted in four headache centers in Serbia. Using a structured questionnaire, we retrospectively studied the prevalence of 16 predefined premonitory symptoms in 321 patients with episodic migraine.

## Results

The mean age of patients was 38.48±12.24 years, 87.9% were women, and 25.8% of them had migraine with aura. At least one premonitory symptom was reported by 263 patients (81.93%). The most frequently reported symptoms were bad mood (61.4%), fatigue (60.7%), irritability (55.7%), stiff neck (55.0%) and concentration problems (54.1%). The mean number of premonitory symptoms per subject was 3.3. Migraine subtype had no effect on the mean number of symptoms per individual, and did not influence the number of symptoms that were always or occasionally associated with migraine attack. Anxiety was significantly more often reported in migraine with aura patients (p = 0.008).

## Conclusions

Premonitory symptoms are frequently reported by migraine patients. Anxiety preceding the attack seems to occur more frequent in migraine with aura.

No conflict of interest.

